# Prevalence of pressure ulcers among hospitalized adult patients in Ethiopia: a systematic review and meta-analysis

**DOI:** 10.1186/s12895-020-00112-z

**Published:** 2020-11-07

**Authors:** Wondimeneh Shibabaw Shiferaw, Yared Asmare Aynalem, Tadesse Yirga Akalu

**Affiliations:** 1 Department of Nursing, College of Health Science, Debre Berhan University, Debre Berhan, Ethiopia; 2 Department of Nursing, College of Health Science, Debre Markos University, Debre Markos, Ethiopia

**Keywords:** Pressure ulcer, Pressure injury, Decubitus ulcer, Bedsore, Ethiopia

## Abstract

**Background:**

Globally, PUs are recognized as one of the five most frequent causes of harm to clients. With millions affected globally, the national pooled prevalence of pressure ulcers in Ethiopia remains unknown. Hence, this review and meta-analysis aimed to determine the prevalence of pressure ulcers among hospitalized clients in Ethiopia.

**Methods:**

Studies were retrieved through search engines in PubMed, Scopus, WHO Afro Library, Google Scholar, Africa Journals Online, and Web of Science. Heterogeneity between-studies were checked using the I^2^ test. A funnel plot and Egger’s regression test was used to assess the presence of publication bias. The random-effect model was fitted to estimate summary effects and 95% confidence intervals (CIs) across studies. The analyses were performed using STATA™ Version 14 software.

**Results:**

The pooled prevalence of pressure ulcer in Ethiopia was assessed using seven studies involving a total of 1881 participants. The pooled prevalence of pressure ulcers in Ethiopia was 11.7% (95% CI: 7.28, 16.13). The subgroup analysis showed that the estimated magnitude of pressure ulcers was 15.89% (95% CI: 13.32, 18.46); among studies, their sample size was greater than or equal to 250.

**Conclusion:**

The current review reported that the pooled prevalence of pressure ulcers in Ethiopia was relatively high. Hence, policymaker and healthcare providers should give attention to reduce the magnitude of pressure ulcers. Furthermore, further a meta-analysis study could be conducted to identify individual and health care service-related factors related to the occurrence of pressure ulcers.

## Background

Pressure ulcers (PUs) primarily occur in bony areas of the body where the potential for pressure and tissue distortion is greatest. Pressure ulcers vary in size and severity of damage to the skin, underlying tissues, muscles, and surface area over bony prominence [1, 2]. Globally, PUs are recognized as one of the five most frequent causes of harm to clients [3]. Although pressure injuries are mainly preventable problems, it has serious impacts on the health care system [4]. It extremely threatens the safety of clients by increasing mortality rates, decreasing quality of life, longer hospital stays, and increasing costs for patient care [5, 6]. Likewise, it impacts pain/suffering, disturbance of body image, delayed healing, and overall health outcomes [4, 7].

In addition to its impact on individual and health care systems, it carries a significant economic burden [8]. It has been estimated that the cost of treating pressure ulcers is 2.5 times higher than the cost of preventing ulcers [9]. The total annual cost for the treatment of PUs in the United States in 2018 was 10.2 billion USD and linked to nearly 29,000 hospital deaths [10].

A meta-analysis revealed that the overall global prevalence of PU using point prevalence was 14.8% [11], with a corollary review in acute care settings yielding rates of between 6 and 18.5% [12]. A meta-analysis reporting on the incidence of PU presentations in emergency departments was 6.31% [13]. Such base knowledge of the PU prevalence rates assists in identifying the severity of the problem, designing preventive strategies, and enhancing the efficient and effective use of healthcare resources [14]. Furthermore, it would be baseline data for quality indicators to measure health care delivery within clinical settings [15].

Substantial variation in the prevalence of pressure ulcers among hospitalized patients across the globe has been seen in the evidence, such as 14.9% in Sweden [16], 18.2% in Norway [17], 10.1% in São Paulo, Brazil [18], 1.58% in China [19], 3.3% in Turk [20], 18.7% in Brazil [21], 17.23% in a sub-Saharan tertiary care center [22], 3.22% in Southwest Nigeria [23], and 19.3% in Tunisia [24].

Evidence showed that multiple risk factors are responsible for PUs [25]. Contributing factors are often associated with the patient’s condition, health care providers, and health care delivery system [26]. A review of several studies on PU clinical risk factors found that immobility [5, 16, 27–29], longer length of hospital stay [16, 27, 29–32], older age [16, 27, 33], reduced sensory perception [16, 30, 33, 34], fecal and urinary incontinence [33, 35], lower Braden scores [6, 27, 33, 36–38], comorbidities [6, 27], and compromised nutritional status [28, 34, 37, 39, 40] were all found to be statistically significant. However, repositioning would reduce the magnitude of pressure over vulnerable areas of the body [1].

The identification of associated factors is a significant consideration in decreasing the risk and incidence of PU [41]. In addition, determining risk factors can be used as yardsticks to design appropriate prevention measures, and to reduce the progression of the disease [42]. Moreover, preventive measures are generally divided into four main areas: assessment of pressure ulcer development risk, skincare and initial treatment, use of pressure-reducing support surfaces, and education [43]. Therefore, early detection of patients who are susceptible to PU is crucial, with the recommendation that an initial skin assessment should be performed within 8 h of hospital admission [44].

Although attention to PU prevention is low in Ethiopia, PU concerns are significant and a major issue in nursing care. Prevention of PU is a key role of nurses and is considered a quality indicator of nursing care [43]. Despite extensive data in developed contexts, there is no comprehensive PU prevalence report in developing contexts, such as Ethiopia. Therefore, the present meta-analysis assessed the prevalence of pressure ulcers among adult hospitalized clients in Ethiopia. Results from the current review could serve as an input for further PU studies in Ethiopia.

## Methods

### Design and search strategy

This systematic review and meta-analysis were carried out by using the Preferred Reporting Items for Systematic Reviews and Meta-Analyses (PRISMA) guidelines [45]. Initially, databases including MEDLINE (via PubMed), Google Scholar, African Journals Online, Scopus, Web of Science, and WHO Afro Library were systematically searched to identify relevant studies electronically. Besides, to identify additional relevant articles, manual search of grey literature available on local university shelves, and institutional repositories was conducted. The current review and meta analysis included articles published from January 1, 2000, to June 1, 2019. To collect and organize search outcomes Endnote X 8.1 reference manager software was used. The keywords used for the review included “pressure ulcer”, “pressure injury”, “decubitus ulcer”, “bedsore”, and “Ethiopia”. To combine search terms Boolean operators such as “AND” and “OR” were used.

### Inclusion criteria

Only articles on research conducted in Ethiopia were included. Studies were eligible for inclusion in the review if they reported their outcome variable as the prevalence of PU. Other criteria were that participants were 18 years of age or older and the research design was quantitative. Similarly, articles published in peer-reviewed journals and gray literature in the English language until June 1, 2019, were also included. Furthermore, the date of publication for this literature search was from January 1, 2000, to June 1, 2019.

### Exclusion criteria

Studies were excluded if: (1) those articles that were not fully accessible; (2) patients admitted with PU; (3) articles in which fail to estimate the outcome (PU); and (4) they have a poor quality score.

### Outcome measurement

This review considered studies that included all stages of PU as their outcome measure. A pressure ulcer was defined as a lesion of the skin or underlying tissues caused by direct unrelieved pressure on the skin. Similarly, according to the European Pressure Ulcer Advisory Panel (EPUAP), PU prevalence is “a cross-sectional count of the number of cases at a specific point in time or the number of people with pressure ulcers who exist in a patient population at a given point in time” [46].

### Data extraction

After identifying articles for inclusion, two authors performed data extraction. For each eligible article, we extracted data regarding the names of the authors, year of publication, study health institution, study design, sample size, sampling technique, outcome assessment, risk assessment tool, and prevalence, plus the quality score of each study was ascertained for each article. Any disagreements at the time of data abstraction were reconciled by discussion and consensus.

### Quality assessment

The methodological quality of each included study was assessed using the Joanna Briggs Institute (JBI) quality appraisal checklist for studies reporting prevalence and incidence studies [47]. This scale has several key criteria to appraise cross-sectional studies, including the following: [1] sample frame appropriate to address the target population, [2] study participants recruited appropriately, [3] adequate sample size, [4] study subjects and setting, [5] data analysis conducted with sufficient coverage of the identified sample, [6] valid methods used for the identification of the condition, [7] outcome measured in a standard, reliable way for all participants, [8] appropriate statistical analysis, and [9] response rate. Studies were considered high quality when scored at 50% and above on the quality assessment indicators. Furthermore, quality assurance checks were independently performed by two authors. Any disagreements at the time of data abstraction were resolved by discussion and consensus.

### Assessment of risk of bias in included studies

The risk of bias of selected articles was assessed using the risk of bias tool for prevalence studies developed by Hoy et al. [48]. After reviewing different studies, the authors decided that articles scoring 8 or more “yes” answers out of a ten-point scale were considered to have a low risk of bias; 5 to 7 “yes” answers were considered to have a moderate risk of bias, and; 4 or fewer “yes” answers were considered to have a high risk of bias.

### Statistical analysis

Data were abstracted by using Microsoft™ Excel, and further analysis was performed using STATA™ Version 14 statistical software [49]. The results of the meta-analysis were reported as the pooled prevalence of pressure injuries with 95% confidence intervals (CIs). Heterogeneity across the studies was evaluated using the I^2^ statistics [50], within a value above 75% interpreted as reflecting high heterogeneity. A random-effects model was performed due to anticipated heterogeneity among studies [51]. To minimize the random variations between the point estimates of the primary study, subgroup analysis was performed based on the study sample size. Besides, to identify the possible sources of heterogeneity, meta-regression was undertaken considering the year of publication and sample size as covariates. Moreover, we performed a sensitivity analysis to describe whether the pooled effect size was influenced by individual studies. However, tests for publication bias were not performed because only 7 studies were included in the analysis [52].

## Result

### Search results

**Fig. 1:**
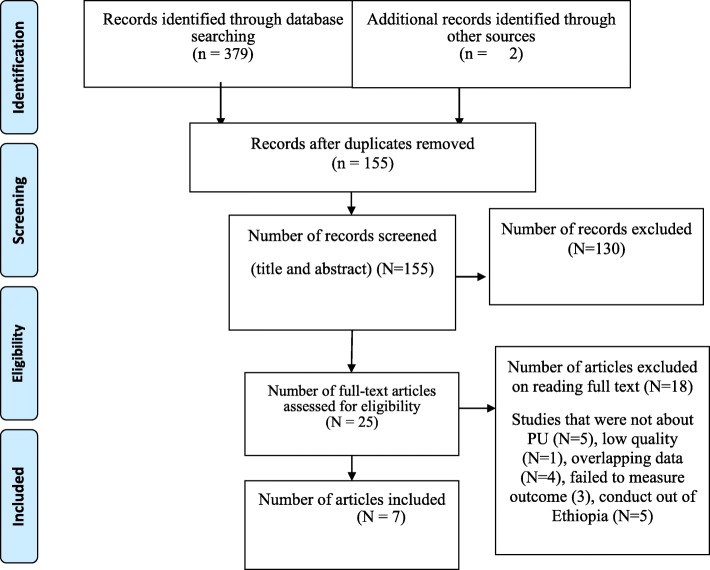
PRISMA flow chart diagram

We found a total of 381 articles based on a systematic international database search, of which 4 articles were found from PubMed, 32 from Scopus, 246 from Google Scholar, 10 from WHO Afro Library, 79 from Web of Science, 8 from African Journals Online, and the remaining 2 were manually searched. Of these, 226 duplicate records were recognized and removed. From the remaining 155 articles, 130 articles were excluded after reading titles and abstracts based on the predefined inclusion criteria. After, 25 full-text articles were evaluated; eventually, merely seven articles were included in the final analysis (Fig. 1).

### Baseline characteristics of the included studies

The pooled prevalence of pressure ulcer in Ethiopia was assessed using seven studies involving a total of 1881 participants. Concerning the region the studies were found, three studies from the Amharic region [30, 40, 53], two from SNNPR [34, 54], one each from Harare [55], and Oromia [38]. Regarding sample size, most (71.4%) of the studies had sample sizes of less than 250. Concerning to study design, all studies were cross-sectional by design (Table 1).

**Table 1:** Baseline characteristics of the included studies

First Author	Publication year	Region	Health Facility Name	Sampling technique	Methods of outcome assessment	Risk assessment tool	Sample Size	Prevalence %(95%CI)
Mengisitie BL et al. [40]	2016	Amhara	Debre Markos Referral Hospital	Systematic random	EPUAP	Braden scale	236	3.4 (1.08–5.71)
Kuruche MM et al. [54]	2015	SNNPR	Wolaita Sodo University Teaching Hospital	Systematic random	EPUAP	Not specified	239	13.4 (9.08–17.7)
Feven T et al. [55]	2016	Harare	Hiwot Fana Haromiya University Hospital	Not specified	Not specified	Not specified	235	16.3 (11.5–21.02)
Bereded D. T et al. [53]	2016	Amhara	Dessie Referral Hospital	Systematic random	EPUAP	Braden scale	355	14.9 (11.2–18.6)
Gedamu H. et al. [30]	2014	Amhara	Felegehiwot Referral Hospital	Systematic random	EPUAP	Not specified	422	16.8 (13.2–20.3)
Ebrahim J. et al. [34]	2016	SNNPR	Hawassa University Referral Hospital	Systematic random	EPUAP	Braden scale	228	8.3 (4.7–11.8)
Assefa T. et al. [38]	2017	Oromia	Jimma University Medical Center	Systematic random	EPUAP	Braden Scale	166	9.6 (5.1–14.08)

### Quality assessment and risk of bias

Based on the Joanna Briggs Institute (JBI) quality appraisal tool, all seven articles fulfilled our predetermined requirement for a score of 6 or higher out of nine total scores (Table 2). On the other hand, a summary of the risk of bias of the included articles showed that six studies (85.7%) were considered to be at low risk of bias [30, 38, 40, 53, 54], while the remaining study was classified as having a moderate risk of bias [34] (Table 3).

**Table 2:** Methodological quality assessment of included studies using the Joanna Briggs Institute (JBI) quality appraisal tool

Corresponding author [reference]	Criteria									
	Appropriate sample frame	Study participants recruitment	Sample size	Study subjects and setting	Data analysis conducted with sufficient coverage	Valid method	Assessment of the outcome	Statistical test	Response rate	JBI score (9)
Mengisitie et al. [40]	Yes	Yes	Yes	Yes	No	Yes	Yes	Yes	Yes	8
Kuruche et al. [54]	Yes	Yes	Yes	Yes	No	Yes	Yes	yes	Yes	8
Feven et al. [55]	Yes	Yes	No	Yes	No	No	Yes	Yes	Yes	6
Bereded et al. [53]	Yes	Yes	Yes	Yes	No	Yes	Yes	Yes	Yes	8
Gedamu et al. [30]	Yes	Yes	Yes	Yes	Yes	No	Yes	Yes	Yes	8
Ebrahim et al. [34]	Yes	Yes	Yes	Yes	Yes	No	Yes	Yes	Yes	7
Assefa et al. [38]	Yes	Yes	No	Yes	Yes	Yes	Yes	Yes	Yes	8

*Note*: From each item account point. (Accept the study if total score ≥ 50%); yes (1 score), no (0 score) from each item

**Table 3:** Risk of a bias assessment tool designed to assess prevalence studies

Corresponding author [reference]	Representation	Sampling	Random selection	non-response bias	Data collected	Case definition	Reliability & validity of tool	Mode of data collection	Prevalence period	Numerator & denominator	The overall risk of bias
Mengisitie BL et al. [40]	No	Yes	No	Yes	Yes	Yes	Yes	Yes	Yes	Yes	Low risk
Kuruche MM et al. [54]	Yes	No	No	Yes	Yes	Yes	Yes	Yes	Yes	Yes	Low risk
Feven T et al. [55]	Yes	Yes	Yes	Yes	Yes	Yes	Yes	Yes	Yes	Yes	Low risk
Bereded D. T et al. [53]	Yes	Yes	No	Yes	Yes	Yes	Yes	Yes	Yes	Yes	Low risk
Gedamu H. et al. [30]	Yes	Yes	Yes	Yes	Yes	Yes	Yes	Yes	Yes	Yes	Low risk
Ebrahim J. et al. [34]	No	No	No	Yes	Yes	Yes	Yes	Yes	Yes	Yes	Moderate risk
Assefa T. et al. [38]	Yes	No	No	Yes	Yes	Yes	Yes	Yes	Yes	Yes	Low risk

*Note*: Risk of bias assessment tool: Yes (low risk); No (high risk): The overall risk of bias scored based on the number of a high risk of bias per study: low risk (≥8), moderate risk (5–7), and high risk (≤4)

### Prevalence of pressure ulcer

**Fig. 2:**
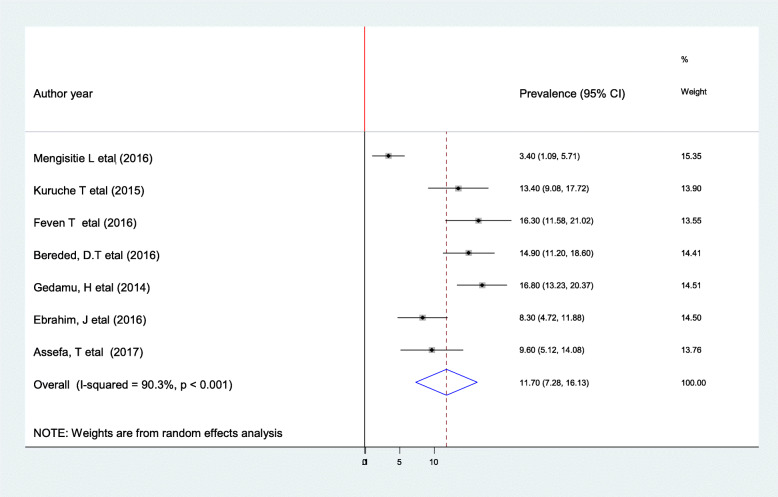
Forest plot showing the pooled prevalence of pressure ulcers in Ethiopia

**Fig. 3:**
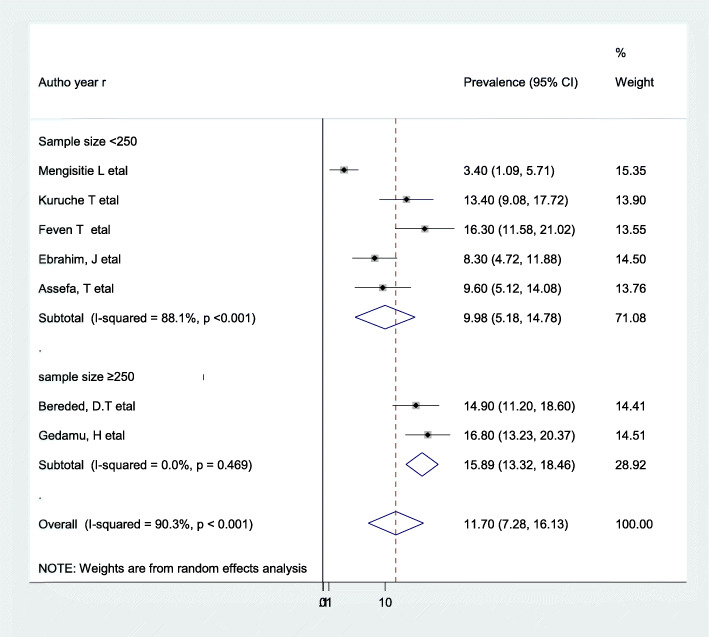
Subgroup analysis by sample size on the pooled prevalence of pressure ulcers

According to the present meta-analysis evidence, the pooled prevalence of PU in Ethiopia was 11.7% (95% CI: 7.18–16.13) (Fig. 2). Using a random-effects model, a statistically significant level of heterogeneity was observed (I^2^ = 90.3%; *p* < 0.001). Due to the presence of significant heterogeneity among the primary, we deployed subgroup analysis by using the study sample size to determine the pooled prevalence of PU. The result of the subgroup analysis showed that the highest prevalence of PUs was reported among study groups whose sample size was greater than or equal to 250, which was 15.89% (95% CI: 13.32,18.46), I^2^ = 0.0% of our included primary studies (Fig. 3).

### Prevalence of pressure ulcer using EPUAP stages

**Fig. 4:**
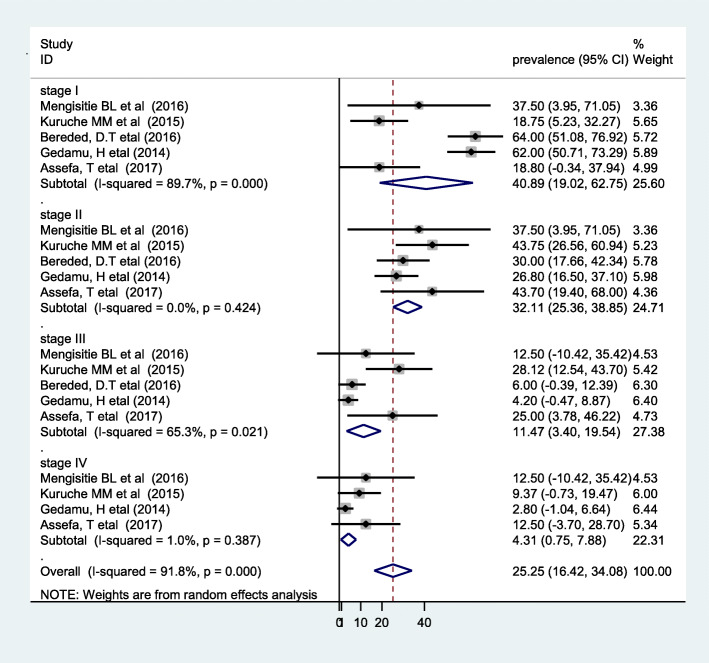
Pooled prevalence of pressure ulcers using EPUAP stages

In the present review, most papers reported pressure ulcers using the EPUAP grading system, while some articles did not report pressure ulcers using the staging system. Based on the European Pressure Ulcer Advisory Panel (EPUAP) grading scale, the pooled estimate of 40.89% developed stage I, 32.11% stage II, 11.47% stage III, and 4.31% stage IV PU (Fig. 4). A summary of the prevalence of pressure ulcers with EPUAP stages is presented in Table 4.

**Table 4:** Prevalence of pressure ulcer using EPUAP stages

Author [reference]	No of the clients develop PU	Prevalence of all stage of PU (%)	Stage I (%)	Stage II (%)	Stage III (%)	Stage IV (%)
Mengisitie BL et al. [40]	8	3.4	37.5	37.5	12.5	12.5
Bereded D. T et al. [53]	53	14.9	64	30	6	–
Gedamu H. et al. [30]	71	16.8	62	26.8	4.2	2.8
Assefa T. et al. [38]	16	9.6	18.8	43.7	25	12.5
Kuruche MM et al. [54]	32	13.4	18.75	43.75	28.125	9.375

*Note***:** The prevalence reports using EPUAP stages (stage I to stage IV) are from the prevalence of all stages instead of the total sample

### Meta-regression analysis

As the test statistic shows, there was significant heterogeneity within and between the included studies (I^2^ = 90.3%). Hence, to identify the possible sources of heterogeneity, we performed meta-regression using publication year and sample size as continuous variables of each article as covariates of interest. But, findings revealed that the publication year and sample size were not statistically significant for the presence of heterogeneity (Table 5).

**Table 5:** Meta-regression analysis for the included studies to identify sources of heterogeneity

Variable	Coefficient	Standard error	t –value	P > |t|	95% CI
Publication year	−0.0058	1.3285	0.00	0.997	(−3.69, 3.680)
Sample size	0.00038	0.0140	0.03	0.979	(−0.038, 0.039)

### Sensitivity analysis

**Fig. 5:**
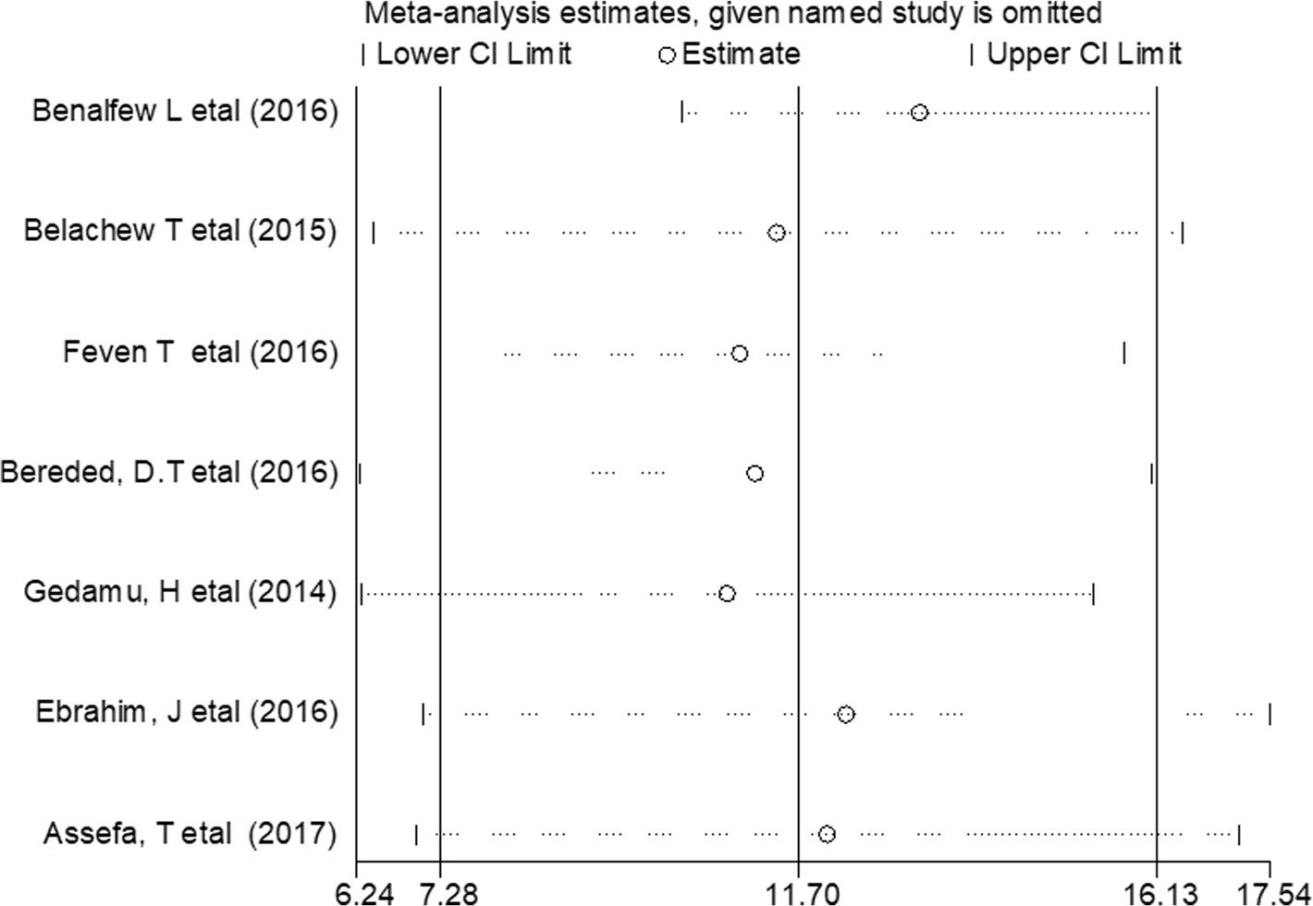
Results of the sensitivity analysis of the 7 studies

The result of sensitivity analysis showed that no single study affected the pooled prevalence of pressure ulcers (Fig. 5).

### Publication bias

In the present review, only 7 studies were included. Therefore, we did not attempt to test for publication bias because test power is usually too low to distinguish chance from real asymmetry.

## Discussion

The current meta-analysis revealed that the pooled prevalence of PU in Ethiopia was 11.7% (95% CI: 7.28, 16.13%). The result of the present meta-analysis is in line with other meta-analysis studies performed on the global prevalence of PU at 14.8% [11], with the findings of a systematic review in acute care settings yielding findings between 6 and 18.5% [12]. On the other hand, our finding is higher than studies done in China (1.58%) [19], Turkey (3.3%) [20], and Southwest Nigeria (3.22%) [23], whereas higher rates were found in a sub-Saharan tertiary center (17.23%) [22], Norway (18.2%) [17], Brazil (18.7%) [21], and Tunisia (19.3%) [24]. The possible explanations for the variations might be methodological differences (i.e., data analysis and eligibility of study participants), variations in the quality of care, and educational preparation among health care providers, as well as policy and strategy differences. Other plausible reasons for the observed difference between the pooled estimates could be variation in sampling and the tool used for assessing the pressure injuries.

In the current meta-analysis, we performed a sub-group analysis based on the study sample size (i.e.sample size < 250 and ≥ 250). As a result, the findings of the subgroup analysis revealed that variability was observed in the overall pooled prevalence across the category of each sample size. Among the category of sample size, the highest pooled prevalence of PU was observed from those studies where the sample size was greater than or equal to 250, which revealed 15.89% (95% CI: 13.32, 18.46). Also, the observed high heterogeneity was explored by publication year and sample size using a meta-regression analysis, although the results did not show any statistical significance. A possible explanation for this variation might be that increasing the sample size would provide a true estimate of the effect.

The current study has implications for clinical practice. This finding would serve as a benchmarks for health care providers to establishing robust preventive measures for averting PUs in hospitals. The finding would enable for nursing educators to facilitate and encourage knowledge of the prevention strategy of PUs among their students to embed this practice as a standard of care. Moreover, the findings serve as input to design and implement different strategies on clients to minimize the burden of PUs across the health care system.

The current meta-analysis is not free from limitations. First, it lacks national representativeness, as no data were found for all regions of Ethiopia. Second, this study did not identify the predictors of pressure ulcers. Third, many of the included studies did not report baseline sociodemographic characteristics of the participants, which prevented subgroup analyses from estimating the prevalence of PU using each variable. Fourth, all included studies reported hospital-based populations, so this review did not consider home-dwelling people with PU.

## Conclusion

The overall pooled prevalence of PU in Ethiopia was relatively high based on the seven evidence-based papers included in this study. Further meta-analysis studies may consider individual and health care service-related factors to the occurrence of PU. A more comprehensive consideration of the existing evidence will potentially inform effective strategies for preventing PU within the Ethiopian context.

## Data Availability

All relevant data are within the paper and its supporting information files. There is no separate data set to share.

## Abbreviations

CI: Confidence Interval
OR: Odds Ratio
PRISMA: Preferred Reporting Items for Systematic Reviews and Meta-Analyses
SNNPR: Southern Nations, Nationalities, People, and region
